# Effects of Single Nucleotide Polymorphisms on Human N-Acetyltransferase 2 Structure and Dynamics by Molecular Dynamics Simulation

**DOI:** 10.1371/journal.pone.0025801

**Published:** 2011-09-29

**Authors:** M. Rajasekaran, Santhanam Abirami, Chinpan Chen

**Affiliations:** 1 Institute of Biomedical Sciences, Academia Sinica, Nankang, Taipei, Taiwan, Republic of China; 2 Department of Life Science, National Tsing Hua University, Hsinchu, Taiwan, Republic of China; 3 Chemical Biology and Molecular Biophysics, Institute of Biological Chemistry, Taiwan International Graduate Program, Academia Sinica, Nankang, Taipei, Taiwan, Republic of China; 4 Institute of Biological Chemistry, Academia Sinica, Nankang, Taipei, Taiwan, Republic of China; 5 Institute of Biochemical Sciences, College of Life Sciences, National Taiwan University, Taipei, Taiwan, Republic of China; University of Akron, United States of America

## Abstract

**Background:**

Arylamine N-acetyltransferase 2 (NAT2) is an important catalytic enzyme that metabolizes the carcinogenic arylamines, hydrazine drugs and chemicals. This enzyme is highly polymorphic in different human populations. Several polymorphisms of NAT2, including the single amino acid substitutions R64Q, I114T, D122N, L137F, Q145P, R197Q, and G286E, are classified as slow acetylators, whereas the wild-type NAT2 is classified as a fast acetylator. The slow acetylators are often associated with drug toxicity and efficacy as well as cancer susceptibility. The biological functions of these 7 mutations have previously been characterized, but the structural basis behind the reduced catalytic activity and reduced protein level is not clear.

**Methodology/Principal Findings:**

We performed multiple molecular dynamics simulations of these mutants as well as NAT2 to investigate the structural and dynamical effects throughout the protein structure, specifically the catalytic triad, cofactor binding site, and the substrate binding pocket. None of these mutations induced unfolding; instead, their effects were confined to the inter-domain, domain 3 and 17-residue insert region, where the flexibility was significantly reduced relative to the wild-type. Structural effects of these mutations propagate through space and cause a change in catalytic triad conformation, cofactor binding site, substrate binding pocket size/shape and electrostatic potential.

**Conclusions/Significance:**

Our results showed that the dynamical properties of all the mutant structures, especially in inter-domain, domain 3 and 17-residue insert region were affected in the same manner. Similarly, the electrostatic potential of all the mutants were altered and also the functionally important regions such as catalytic triad, cofactor binding site, and substrate binding pocket adopted different orientation and/or conformation relative to the wild-type that may affect the functions of the mutants. Overall, our study may provide the structural basis for reduced catalytic activity and protein level, as was experimentally observed for these polymorphisms.

## Introduction

The enzymes arylamine N-acetyltransferases (NATs, EC 2.3.1.5) catalyze the transfer of an acetyl group from acetyl CoA to arylamine, hydrazines and their *N-*hydroxylated metabolites, thus playing an important role in detoxification and potential metabolic activation of numerous xenobiotics [1]. NATs have been identified and characterized in many eukaryotes and prokaryotes [2]. In humans, NAT exists as 2 isozymes: NAT1 and NAT2, both of which are 290 amino acids long and share 81% sequence identity but differ in their tissue distribution and substrate specificity [3]. Both NAT1 and NAT2 are highly polymorphic and catalyze the metabolism of many aromatic amines and hydrazine drugs. NAT2 mainly catalyzes the *N*-actetylation of arylamines and the *O*-acetylation of *N*-hydroxylated metabolites of arylamines and heterocyclic amines [4]. Genetic variability in NAT2 is responsible for the variation in the inactivation of the anti-tubercular drug isoniazid [5]. NAT2 acetylation polymorphism modifies the toxicity of a number of aromatic and hydrazine drugs in humans [6].

To date, 34 single nucleotide polymorphisms (SNPs) have been identified in the human NAT2 protein coding region (http://louisville.edu/medschool/pharmacology/consensus-human-arylamine-n-acetyltransferase-gene-nomenclature/nat_pdf_files/Human_NAT2_alleles.pdf). According to the consensus of gene nomenclature of human NAT2, 62 NAT2 alleles or haplotypes are characterized by different combinations of these SNPs. Allelic variations in NAT2 result in rapid, intermediate and slow acetylator phenotypes [7], [8], [9]. The NAT2 allele (named NAT2*4), classified as a wild-type (WT) allele with no SNPs, encodes an NAT2 protein with high *N*-acetylation activity and confers a rapid acetylator phenotype [10]. The SNPs in NAT2 such as G191A (R64Q), T341C (I114T), G364A (D122N), A411T (L137F), A434C (Q145P), G590A (R197Q), and G857A (G286E) are associated with slow acetylator phenotypes. The amino acid substitution in these mutants (MTs) results in a low metabolic rate of aromatic and heterocyclic amines. The decreased catalytic activity of slow acetylators leads the arylamines to be shunted toward the hydroxylation pathway, which results in DNA reactive metabolites [11]. Slow acetylators are associated with a high risk of cigarette smoking-related breast cancer [11] and urinary bladder cancer [12]. Some epidemiological studies have reported that NAT2 polymorphisms modify the individual risk of various cancers such as colon cancer [13], breast cancer [14], [15], [16] and prostate cancer [17], [18] with carcinogen exposure. Individuals with slow acetylator phenotypes show a reduced amount of NAT2 protein expression in human liver [19], [20]. Some of the slow acetylator recombinant NAT2 proteins show reduced stability [7], [21], [22] and others are targeted for proteosomal degradation [23], [24]. Certain individual SNPs such as G191A (R64Q), G364A (D122N), A434C (Q145P) and G590A (R197Q) have reduced catalytic activity, whereas G857A (G286E) differs in efficiency for different substrates [10].

To understand the key structural changes induced by SNPs, the homology modeled structure of NAT2 has been investigated in a few studies [25], [26], [27]. These studies mainly used prokaryotic NAT2 as a template to build a human NAT2 model. However, the sequence similarity between the bacterial and human NAT2 is low (∼30%), and human NAT2 possesses an extra 17-residue insertion region. These differences limit the use of the human NAT2 model in understanding the effect of SNPs on the NAT2 structure.

**Figure 1:**
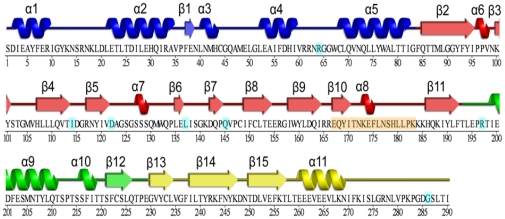
Sequence and secondary structure of N-acetyltransferase 2 (NAT2). The secondary structures are shown on the top of the sequence and the residue numbers are shown on the bottom of the sequence. Each domain is colored separately (Domain 1-blue, Domain 2-red, Inter Domain-green and Domain 3-yellow). The single nucleotide polymorphism (SNP) residues and the 17-residue insert region are highlighted in cyan and orange respectively.

**Figure 2:**
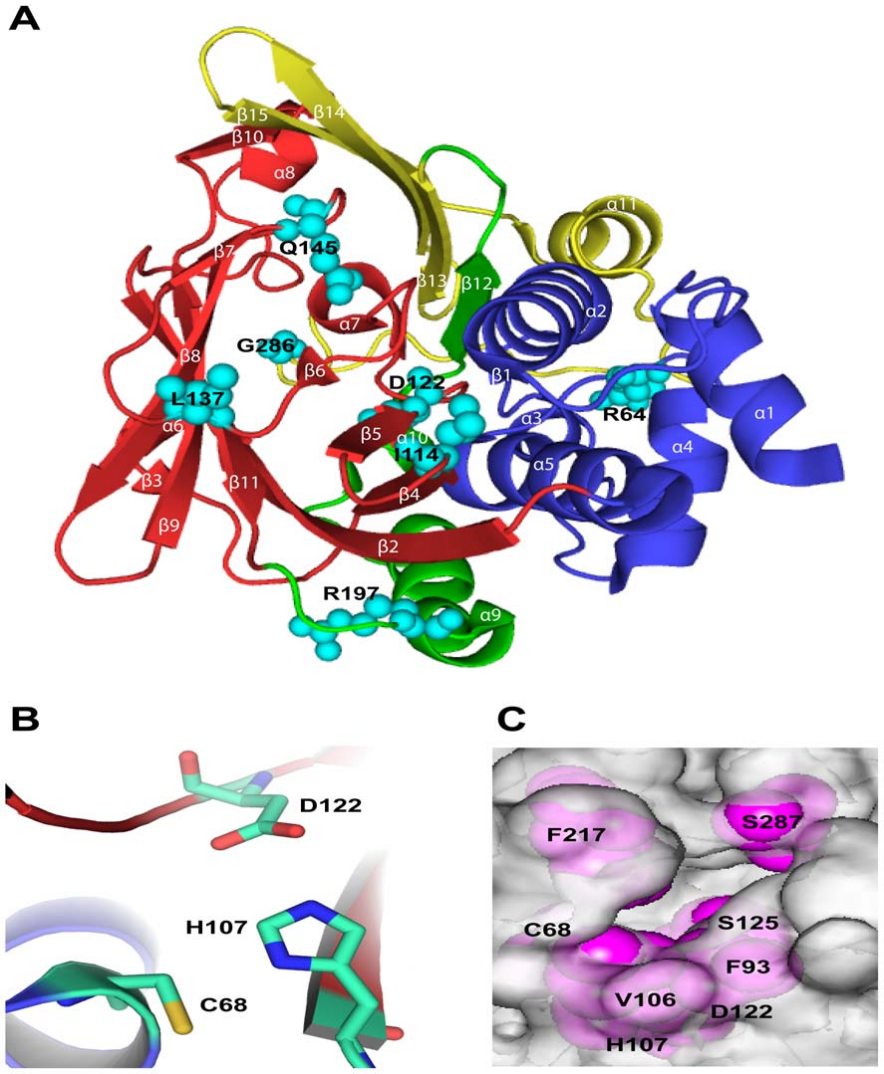
Structure of N-acetyltransferase 2 (NAT2). (A) The overall fold of human NAT2 (PDB code: 2PFR) is shown in cartoon representation, and each domain is colored as in Figure 1. The SNP residues are shown as space-filling representation in cyan. (B) The orientation of catalytic triad residues C68, H107 and D122 is shown in stick representation, with carbon atoms in cyan, nitrogen in blue, oxygen in red and sulfur in yellow. (C) Molecular surface in the vicinity of the substrate binding pocket of WT. The residues forming the pocket are in magenta.

Recently, the high resolution structure of human NAT2 was determined [28] . Residues S1 to G83 in the N-terminus mainly consisting of α-helices form a domain 1 (D1); residues F84-F192 mainly consisting of β-strands form a domain 2 (D2) followed by inter-domain (ID) comprising residues T193-E229 and residues G230 to I290 consisting of both α-helix and β-strands form a domain 3 (Figure 1 and 2A). Similar to prokaryotic NATs, human NAT2 also contains a cysteine protease-like catalytic core, consisting of C68, H107 and D122, (Figure 2B), and the arrangement of these catalytic residues is maintained by a hydrogen-bond (H-bond) network and non-bond contacts. The 17-residue insertion is absent in the prokaryote NATs, and the exact function of this insertion is not clear, although one study suggests that it may contribute to the stability of the human NATs [27]. Also, C-terminus of human NATs extends deep into the active site, which is not observed in the prokaryote NATs. Previous studies have proposed that NATs catalyze acetyl transfer by a ping-pong bi-bi mechanism [29], [30]. The formation of a thiolate-imidazolium ion pair by C68 and H107 was found to be catalytically important. The other catalytic triad residue D122 involved in ionic interaction with H107 is presumably important for the activity and the structural integrity [31]. The substrate-modeled crystal structure shows that the substrate fits well into substrate binding pocket (SBP) [28], which is mainly formed by residues C68, F93, V106, H107, D122, S125, F217 and S287 (Figure 2C). NAT proteins catalyze acetyl CoA (cofactor) dependent acetylation of the substrates so that the binding of cofactor is an important step in the NAT protein catalysis process. However in the reported crystal structure, NAT2 binds with CoA (Figure S1A), not acetyl CoA. Based on this crystal structure, Oda *et al* [32] modeled the structure of NAT2 in complex with the acetyl CoA (Figure S1B), and found that the hydrophobic core comprising F37, W67, L69, F93, I95, V98, V106, F202, L209, F217, and L288 around the SBP plays an important role in the binding of acetyl CoA. The SNPs in this study distribute throughout the structure and except for D122N, the remaining are ∼12 to 17 Å away from the catalytic site.

Walraven *et al* [10] evaluated the SNP effect on the basis of the NAT2 crystal structure, especially the H-bond interaction pattern at the mutational site. However, the other changes, such as conformational change at the catalytic triad, changes at the SBP, dynamic motion of different regions and surface charge property of the MTs, which are important for understanding the structural basis related to the reduced catalytic activity and degradation of the MTs, were not explained in detail. To completely understand the effect of these polymorphisms at atomic resolution, the high-resolution structures of the NAT2 variants are needed. However, most of the NAT2 variants result in increased degradation or aggregation, which makes structural studies difficult.

An atomic-level look at the protein dynamics through multiple molecular dynamics simulations can help understand the effects of these mutations on the protein structure, which allows for investigating how one amino acid change can create a ripple effect throughout the protein structure and eventually affect cofactor binding, substrate binding, enzyme activity and protein degradation. In this study, we generated the structures of 7 mutants on the basis of the NAT2 crystal structure and investigated them and the apo wild type (WT) by multiple molecular dynamics simulation. These mutations changed the surface property and induced structural changes that propagated through space to distort the orientation of the catalytic residues, cofactor binding site, SBP and especially the flexibility of domains ID and D3. Residues E174-L181 of the 17-residue insert showed reduced flexibility in all 7 MTs. Our results provide possible explanations for the decreased catalytic activity and degradation of the SNPs.

## Materials and Methods

### Starting structure

The bound coenzyme A and sulphate ion from the 1.92 Å crystal NAT2 structure (PDB code: 2PFR) were removed, and the resulting structure with 5000 steps (2000 steps of steepest descent and 3000 steps of conjugated gradient) of energy minimization was used as the starting structure of WT NAT2. The starting structures for MTs were generated by substituting the amino acids for the WT protein using Discovery studio 2.5 software, and then performing 5000 steps of energy minimization.

### Molecular dynamics simulation

Molecular dynamics simulations of WT and MTs were performed using GROMACS [33] that adopts the GROMOS96 force-field parameters for energy minimization and molecular dynamics simulations [34]. All the structures were solvated in a periodic cubic box with walls extending at least 10 Å from all atoms. To obtain an electrically neutral system, the GENION procedure from the GROMACS package was used to replace water molecules with Na^+^ and Cl^−^ ions. The solvated structures were energy minimized with the steepest descent method for 200 ps without using positional restraints. Then, 600 ps of molecular dynamics with position restraints were performed to gently relax the system, followed by dynamics simulations of the full system without any positional restraints for 40,000 ps. Three independent simulations of each protein were performed for a total of 960 ns of simulation time.

All the simulations were performed in the NPT ensemble at a constant temperature (310 K), and the pressure was maintained by coupling to a reference pressure of 1 bar. The non-bonded pair list was updated every 10 steps. The PME algorithm [35] was applied to treat electrostatic interactions. All the bonds were constrained with use of the LINCS algorithm [36], and the SETTLE algorithm [37] was used to constrain the geometry of the water molecules. A time step of 2 fs was used in all calculations, and coordinates were saved at regular time intervals of every 1 ps.

### Analysis of molecular dynamics trajectories

Structural properties, such as root mean-square deviation (RMSD) and root-mean square fluctuation (RMSF), were calculated with the built-in functions of GROMACS. The solvent accessible surface area (SASA) was calculated with the NACCESS algorithm [38]. The hydrogen bond interactions were calculated with the HBPLUS program [39]. All these analyses involved use of the last 10-ns structures (Figure S2) of each simulation. The electrostatic potential was calculated with the Discovery studio 2.5 and the protein interaction property similarity analysis server (PIPSA) [40], [41], which computes the electrostatic similarity and dissimilarity between the WT and MTs. The last 10-ns averaged structures from one simulation were used to calculate the electrostatic potentials. The server uses the APBS software [42] to calculate the electrostatic potentials, then the Hodgkin similarity indices [43] were calculated for all pairs of proteins based on the electrostatic similarity, then the similarity indices were converted to electrostatic distances. Finally, the distance matrix is displayed in a color-coded heat map form.

## Results and Discussion

### Effects of the mutations on NAT2 global structure

The overall structural parameters from the simulations are in Table 1. To understand the overall structural stability throughout the simulation, we calculated the RMSD of Cα atoms (Cα-RMSD) from the appropriate starting structures for each simulation. For the WT, the mean overall Cα-RMSD is 2.0 Å, whereas that for MTs ranges from 1.9 to 2.2 Å. Relative to the WT, in MTs, the amino acid substitutions do not increase the mean Cα-RMSD, and similarly, all MT structures do not show an increase in total SASA. Therefore, MT structures are stable and do not expand appreciably during simulation. Interestingly, the mean number of total H-bonds during the entire simulation differs slightly between the WT and the MTs R64Q, D122N, L137F, R197Q and G286E. Although the difference is not great, the reduced number of H-bonds may affect the structural integrity. Further, the last ns (40^th^) structures from 3 independent simulations of each protein (Figure S3) indicate that all proteins retain the NAT2 fold topology throughout the simulations.

**Table 1:** General properties of wild-type (WT) and mutant (MT) simulations^a^.

Molecule	Cα-RMSD^b^ (Å)	Total SASA^c^ (Å^2^)	Total number of hydrogen bonds
WT	2.0±0.8	13210.17±147.89	264±7
R64Q	2.1±0.6	12278.23±126.62	249±8
I114T	2.0±0.4	12676.21±142.94	258±6
D122N	2.1±0.4	12234.29±138.44	246±5
L137F	2.2±0.5	12175.64±123.53	249±8
Q145P	2.0±0.3	12745.60±156.89	252±9
R197Q	1.9±0.2	12126.91±112.01	248±5
G286E	1.9±0.4	12616.45±160.51	249±5

a Properties represent averages for 3 simulations over the last 10 ns of each simulation.

b Cα-RMSD - Root mean square deviation from the starting structure.

c SASA - Solvent accessible surface area.

### Effects of the mutations on NAT2 structural flexibility

**Figure 3:**
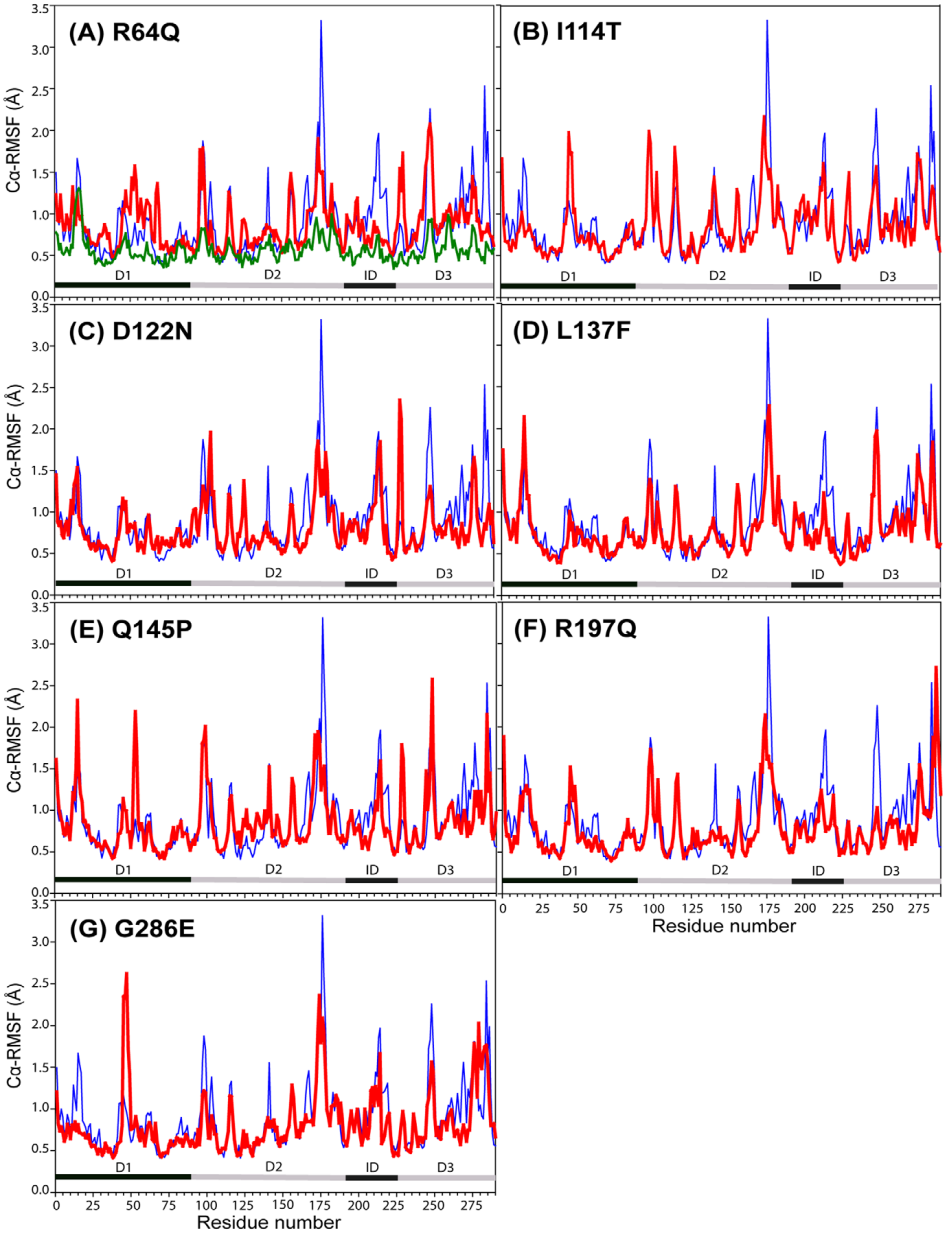
Flexibility of WT and MT structures. The figure shows Cα-RMSF values (in Å) per residue for the WT (blue) compared with MTs (A) R64Q, (B) I114T, (C) D122N, (D) L137F, (E) Q145P, (F) R197Q and (G) G286E (all in red). Crystallographic *B*-factors from the WT structure (PDB code: 2PFR) are colored in green. The Cα-RMSF values were calculated from the last 10-ns structures of each simulation. The alternate black and gray lines at the bottom of the figure indicate the different domains.

To understand how these mutations affect the overall flexibility of the protein, we calculated the RMSF of Cα atoms (Cα-RMSF), which reflects the degree of main-chain fluctuations from the mean structure over the simulation (Figure 3A-G). In the WT simulations, Cα-RMSF values are almost on the same scale and pattern as those of NAT2 crystallographic B-factors. Compared with WT, all the MTs have specific regions with increased or decreased fluctuations, specifically, the 17-residue insert region ID and D3 showing a reduced flexibility in all MTs. Further, analysis of the residues with >1 Å Cα-RMSF (Table 2) revealed that both ID and D3 have lower percentage of residues, which indicates that these 2 domains are more rigid. All MTs possessed a common behavior in flexibility changes in the 17-residue insert region, ID and D3.

**Table 2:** Percentage of residues in each domain with >1 Å Cα-RMSF value calculated in WT and MT simulations.

Molecule	D1^a^	D2^b^	ID^c^	D3^d^
WT	12.8	18.4	28.5	35.0
R64Q	13.3	15.4	11.2	21.1
I114T	9.3	20.1	23.2	28.3
D122N	9.4	15.2	18.6	12.8
L137F	9.1	14.5	7.4	20.2
Q145P	14.3	19.4	12.3	21.4
R197Q	12.5	16.2	8.1	15.2
G286E	8.7	15.3	22.1	26.5

a D1 - Domain 1 (residues 1–83).

b D2 - Domain 2 (residues 84–192).

c ID - Inter-domain (residues 193–229).

d D3 - Domain 3 (residues 230–290).

### Hydrogen bond changes at the mutational site

The amino acid substitutions in MTs are expected to result in certain H-bond changes around the mutational site. We found no apparent change in H-bond at the mutational site for I114T, L137F and G286E, whereas some of the important H-bonds were lost in R64Q, D122N, and Q145P (Table 3). For example, R64 of the WT forms H-bonds with N41, H58 and I59 and a salt bridge with E38, and these interactions were broken in R64Q. Because N41 and E38 are highly conserved in NATs, the H-bond (R64/N41) and the salt bridge (R64/E38) are important for protein function and/or stability [10]. E38 also forms an H-bond with Q64 in R64Q but has a low percentage of occupancy, and the interaction of the salt bridge is stronger than that of the H-bond, so this H-bond may not be critical in function and stability of the protein, as is the salt bridge in the WT. D122 of the WT forms an H-bond with N72, G124, S125, Q130 and Y190 and an ionic interaction with H107. In D122N, some of these H-bonds were retained, but the important ionic interaction and the H-bonds with residues S125 and Y190 were lost. Because residues N72, H107, D122 and Y190 are highly conserved in mammalian and prokaryotic NAT2, these residues and their interactions are important for protein function. Although S125 is highly variable, it is important for substrate selectivity [44]. Thus, disruption of the H-bonds affects the conformation of the catalytic triad and substrate binding site, which in turn affects the catalytic activity. In Q145P, the substitution of Pro disrupts the H-bond interactions with W132 and Q133, and loss of these H-bonds increases the flexibility around the mutational site.

**Table 3:** Occupancy percentage of the hydrogen bond interactions at the mutation positions in the WT and MTs.

WT	Hb^a^	Occu^b^	MTs	Hb^a^	Occu^b^
R64	9ARG(NH2):::64ARG(O)	18	R64Q	62ARG(NH2):::64GLN(OE1)	40
	9ARG(NH1):::64ARG(O)	16		46GLN(NE2):::64GLN(OE1)	27
	64ARG(NH2):::38GLU(OE2)	87		46GLN(NE2):::64GLN(NE2)	19
	64ARG(NH2):::38GLU(OE1)	77		64GLN(NE2):::38GLU(OE2)	24
	64ARG(NH2):::41ASN(OD1)	88			
	64ARG(N):::58HIS(O)	34			
	64ARG(NH2):::59ILE(O)	51			
I114	114ILE(N):::117ARG(O)	72	I114T	114THR(N):::117ARG(O)	71
	117ARG(N):::114ILE(O)	45		117ARG(N):::114THR(O)	14
D122	190TYR(OH):::122ASP(OD1)	80	D122N	122ASN(ND2):::108LEU(O)	24
	190TYR(OH):::122ASP(OD2)	36		122ASN(ND2):::196TYR(OH)	76
	130GLN(NE2)::: 122ASP(O)	70		130GLN(NE2):::122ASN(O)	57
	125SER(OG)::: 122ASP(OD1)	97		124GLY(N):::122ASN(OD1)	47
	125SER(N)::: 122ASP(OD1)	25		72ASN(ND2):::122ASN(OD1)	93
	124GLY(N)::: 122ASP(OD1)	54			
	124GLY(N)::: 122ASP(OD2)	88			
	107HIS(NE2)::: 122ASP(OD2)	99			
	72ASN(ND2)::: 122ASP(OD2)	97			
L137	137LEU(N):::118ASN(O)	90	L137F	137PHE(N):::118ASN(O)	35
				139SER(OG):::137PHE(O)	17
Q145	145GLN(NE2):::133GLN(O)	82	Q145P	148CYS(N):::145PRO(O)	14
	145GLN(NE2):::148CYS(O)	59			
	133GLN(N):::145GLN(OE1)	92			
	132TRP(N):::145GLN(OE1)	19			
R197	197ARG(N):::88MET(O)	93	R197Q	197GLN(NE2):::105MET(O)	58
				197GLN(NE2):::201ASP(OD1)	16
				197GLN(N):::88MET(O)	88
				197GLN(OE1):::90GLY(N)	35
G286	-		G286E	-	

a Hydrogen bonds.

b Hydrogen bond occupancy percentage, only occupancies with ≥10 in each simulation are shown.

Therefore, the disruption of important H-bond interactions in R64Q, D122N, and Q145P affects the flexibility around the mutational site, which may affect the protein function.

### Effects of the mutations on NAT2 local tertiary structure

**Figure 4:**
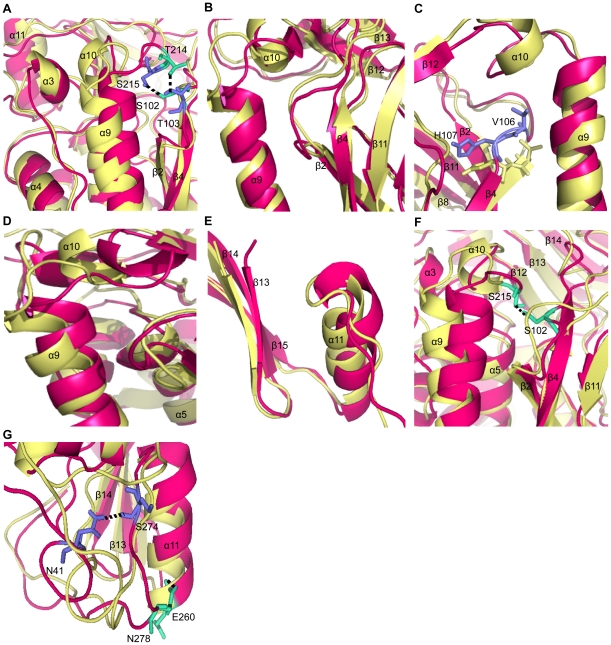
Mutational effects on NAT2 local tertiary structure. The local tertiary structure of the MTs (pink) is superimposed with the WT structure (yellow). The average structures from last 10-ns of one simulation are shown here. The depicted structural changes were observed in all the simulations for each protein. (A) In R64Q, two new H-bonds are formed, which are shown in stick representations. The first one is between T214 (cyan) and T103 (blue) and the other is between S215 (blue) and S102 (cyan), which pull the α9 towards the β2 and β4. (B) In I114T, most of secondary structures conformation is very similar to the WT. (C) In D122N, residues V106 and H107 (blue, stick representation) loose its secondary structure and become more flexible. The WT residues V106 and H107 are in yellow, stick representation. (D) In L137F, steric clash in β sheets affect the orientation of α9 and α10. (E) In Q145P, the D3 (pink) expands slightly as compared with the WT (yellow). (F) In R197Q, one new H-bond is formed, which is shown as cyan, stick representations. The residue S215 in the ID forms H-bond with D2 residue and S102, and pulls the α9 towards β2 and β4. (G) In G286E, the two newly formed H-bonds S274-N41 (blue) and N278-E260 (cyan) significantly alter the conformation of the C-terminal tail relative to the WT.

Although mutations do not affect the global structure, the local tertiary structures are significantly affected. To gain insight into these changes, we analyzed the last 10-ns average structures for each mutant. In R64Q, change in the fluctuation slightly alters the orientation of secondary structures among α3, β2, β4 and β10, and causes a large orientation change in α9 and α11. The formation of new H-bonds by residues T214 and S215 in the α9–α10 loop with T103 and S102 in the β3–β4 loop, respectively, pulls the α9 towards the β2 and β4 (Figure 4A). I114T shows no significant change in orientation of the secondary structure (Figure 4B). In D122N, the flexibility is increased in the β3–β4 loop. Residues V106 and H107 in β4 lose the secondary structure and hence are more flexible, which causes a significantly conformational change of the catalytic triad residue H107 (Figure 4C). In L137F, F137 induces a steric clash with the surrounding β-sheets formed by β2, β4, β5, β7, β8 and β11. This steric clash makes a slightly orientation change in these β-sheets. Especially, the major steric clash is observed between F137 and F191 of β11. To accommodate F137, the β11 slightly alters its orientation, which in turn affects the conformation of the following loop, α9 and α10. Compared with WT, the α9 moves towards the inside, which affects the orientation of the α9-α10 loop and α10 (Figure 4D).

In Q145P, the increase in dynamics motion in D1 affects the adjacent D3, especially in β13, β14 and α11. These regions move outwards and expand the structure in the D3 (Figure 4E). This observation is consistent with the total SASA of the Q145P (∼12745 Å), which is slightly higher than that of the other MTs (Table 1). Therefore, Q145P slightly expands, especially in the region of D3. In R197Q, substitution of Gln for Arg affects the flexibility and orientation of the secondary structures. The most notable change is observed in α9. Formation of a new H-bond by S215/S102 pulls the α9 towards β2 and β4 (Figure 4F), which restricts the flexibility of the α9 and the entire ID. In G286E, the conformation of the C-terminal tail is altered significantly, primarily because of the formation of 2 new H-bonds (S274/N41 and N278/E260) (Figure 4G).

### Effects of the mutations on NAT2 catalytic triad conformation

**Figure 5:**
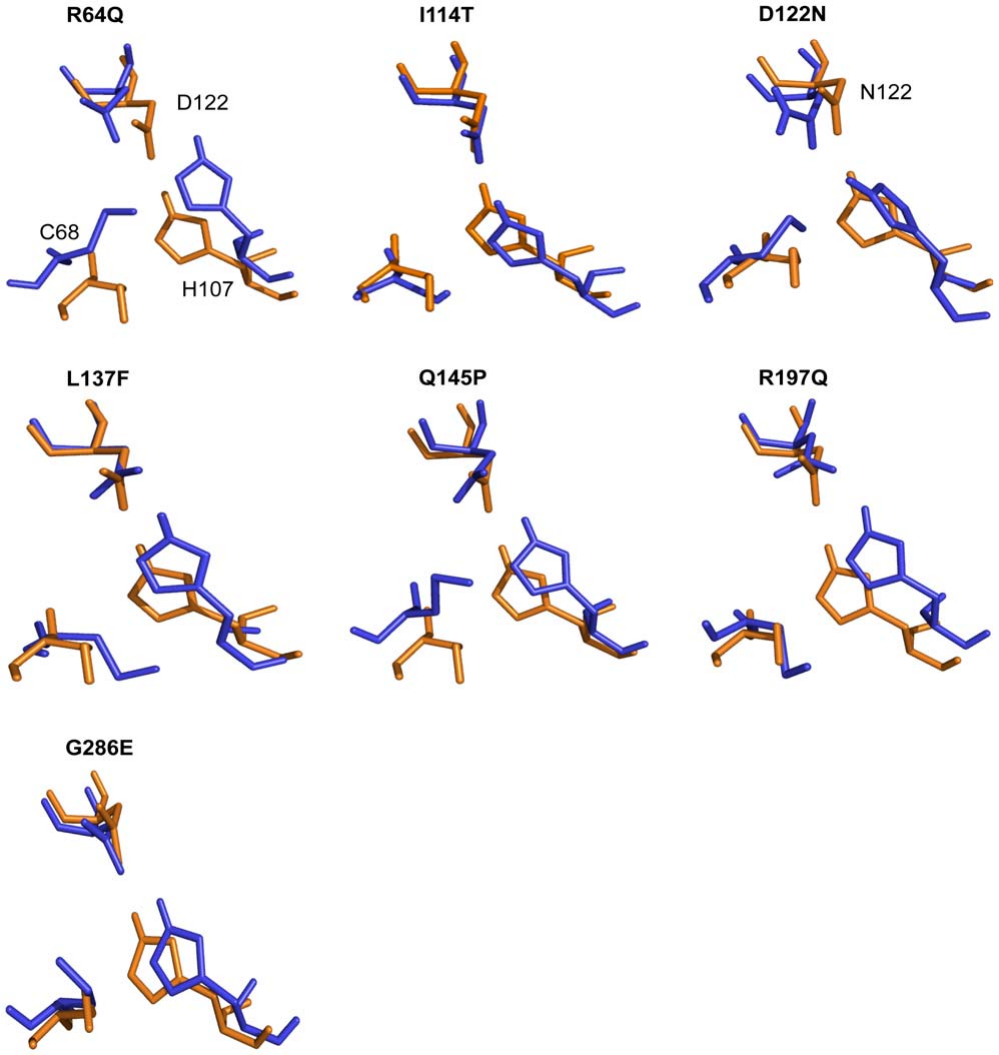
Structural effects of mutations on NAT2 catalytic triad residues from molecular dynamics simulation. The averaged structure from last 10-ns of the simulation of WT (orange) is superimposed with each MT (blue) averaged structure. Similar conformational changes were observed in all simulations for each protein.

To better understand how the mutations induce conformational changes in the catalytic triad, we compared the conformation of the catalytic triad of the WT and MTs (Figure 5). Changes in the local tertiary structure and flexibility significantly affect the conformation of the catalytic triad in all MTs except for I114T and G286E; for both, the conformation and relative orientation of the catalytic triad residues are similar to that of the WT. In addition, the ionic interaction between the H107 and D122 is preserved throughout the simulations for I114T and G286E, whereas in all other MTs, this interaction is not seen. Conformation of these residues is also severely altered, and the increased distance between C68 and H107 may hinder the formation of the thiolate-imidazolium ion pair.

### Effects of the mutations on NAT2 cofactor and substrate binding pocket

**Figure 6:**
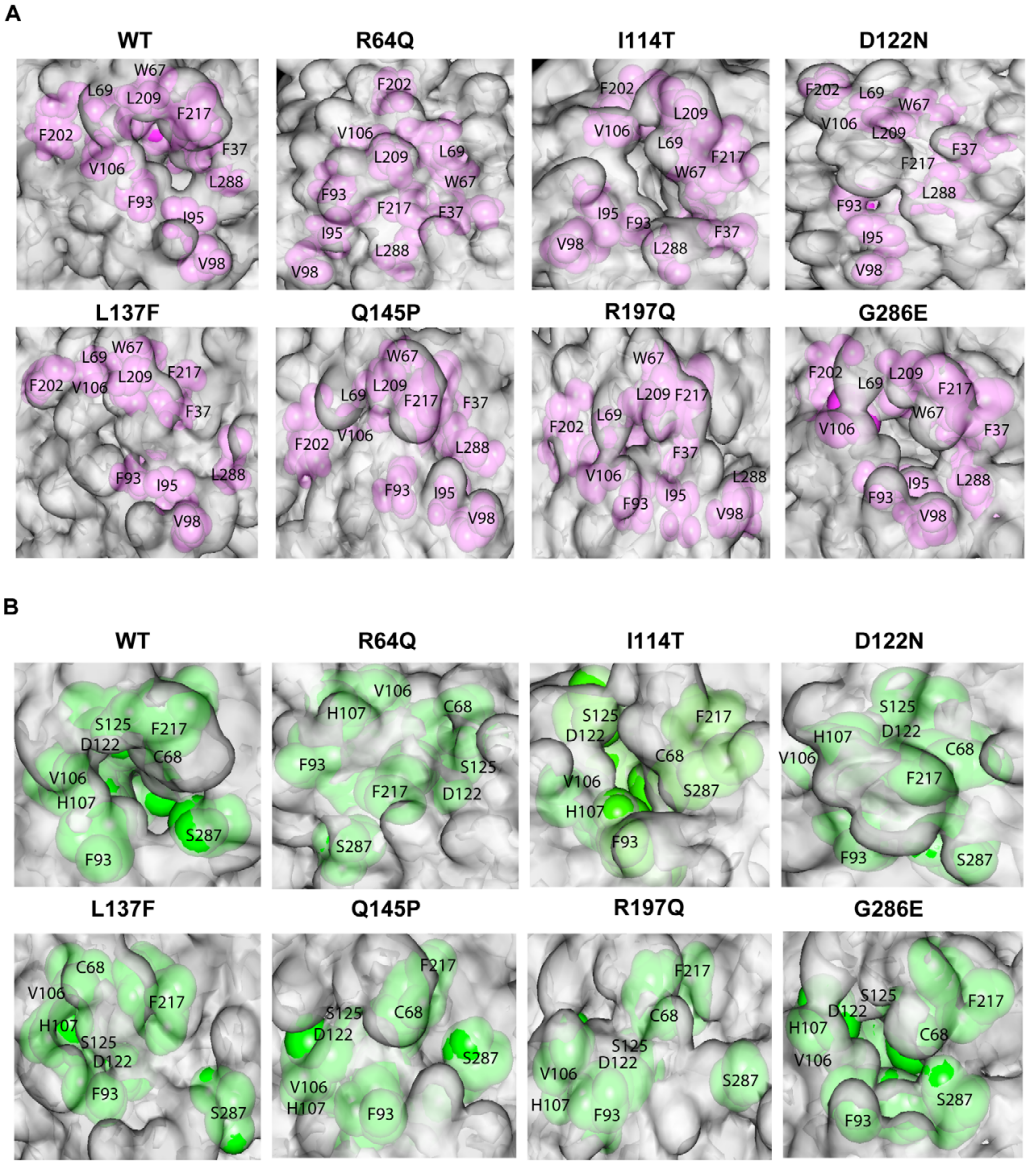
Structural effects of the cofactor binding site and substrate binding pocket on MTs. (A) Molecular surface in the vicinity of the cofactor binding site of the WT and MTs. The surface portions corresponding to the residues involved in forming the hydrophobic core around the substrate binding pocket (F37, W67, L69, F93, I95, V98, V106, F202, L209, F217, and L288) are in magenta. (B) Molecular surface in the vicinity of the substrate binding pocket of the WT and MTs. The surface portions corresponding to residues involved in forming the substrate binding pocket (C68, F93, V106, H107, D122, S125, F217 and S287) are in green. A cavity is observed only in WT, I114T and G286E. Structures were taken from the snapshot at 40^th^ ns from one simulation. Almost similar changes were observed in all the simulations of WT and each MT.

In NAT2, the acetyl CoA molecule acts as a cofactor and plays an important role in the acetylation of the substrates. The proposed reaction mechanism contains 2 steps. In the first step, the acetyl CoA–NAT2 complex is formed and then the acetyl group of acetyl CoA is transferred to the catalytic triad residue C68. Subsequently, the acetyl group from acetylated cysteine is transferred to the substrate. Residues important for SBP and acetyl CoA binding are nearby or overlapped, and those for CoA binding form a hydrophobic core around SBP. Investigating the SASA of the hydrophobic core and SBP revealed that changes in the structure and dynamics greatly affect the hydrophobic core and SBP size and/or shape (Table 4). The SASA values of all MTs, except I114T and G286E, were greatly reduced, so the hydrophobic core and SBP in I114T and G286E are close to those of the WT. For the remaining MTs, the binding residues are more buried, thereby altering the hydrophobic core and SBP. In addition, the surface analysis of hydrophobic core (Figure 6A) and SBP (Figure 6B) showed that for I114T and G286E and the WT, the hydrophobic core and SBP region is retained, although the volume of the pocket is not similar. For other MTs, surface analysis revealed that the hydrophobic core and SBP are significantly distorted, and no pocket is observed. The distortion of hydrophobic core and SBP in these MTs may decrease the binding affinity for cofactor and substrate. Because the mutants of NAT1 are unable to bind the cofactor acetyl CoA, which results in protein degradation [45], NAT2 MTs may also undergo a similar process.

**Table 4:** Solvent accessible surface area (SASA) of the substrate binding pocket (SBP) and hydrophobic core around the SBP in WT and MT simulations^a^.

Molecule	SASA Hydrophobic Core^b^ (Å)^2^	SASA SBP^c^ (Å)^2^
WT	310.76±19.22	160.31±22.35
R64Q	255.42±19.43	124.13±23.66
I114T	298.67±18.54	156.66±24.24
D122N	249.77±19.41	122.55±21.44
L137F	258.22±19.66	126.71±21.23
Q145P	248.71±19.76	128.47±21.45
R197Q	258.88±21.11	124.57±22.75
G286E	291.55±19.76	152.46±22.66

a SASA was calculated using structures from last 10-ns of each simulation.

b The following residues were used to calculate the SASA of the hydrophobic core around the SBP site: F37, W67, L69, F93, I95, V98, V106, F202, L209, F217 and L288.

c The following residues were used to calculate the SASA of the SBP site: C68, F93, V106, H107, D122, S125, F217 and S287.

Functional study revealed reduced protein level and enzymatic activity for R64Q, D122N, L137F, Q145P and R197Q [24]. For I114T and G286E, functional study revealed reduced protein level, but the apparent kinetic parameters or protein stability did not change in I114T [23], and the activity for certain substrates is retained in G286E [7]. Our simulation data revealed that the MTs R64Q, D122N, L137F, Q145P and R197Q affect the flexibility of some domains and alter the orientation of some secondary structures. These changes may be responsible for altering the catalytic triad, hydrophobic core and SBP, which may prohibit the binding of acetyl CoA and substrate, thereby significantly decreasing the catalytic activity in these MTs. For I114T and G286, most of the structural properties are close to that of the WT, which is why both MTs retain the catalytic activity.

### Effects of the mutations on NAT2 electrostatic potential

**Figure 7:**
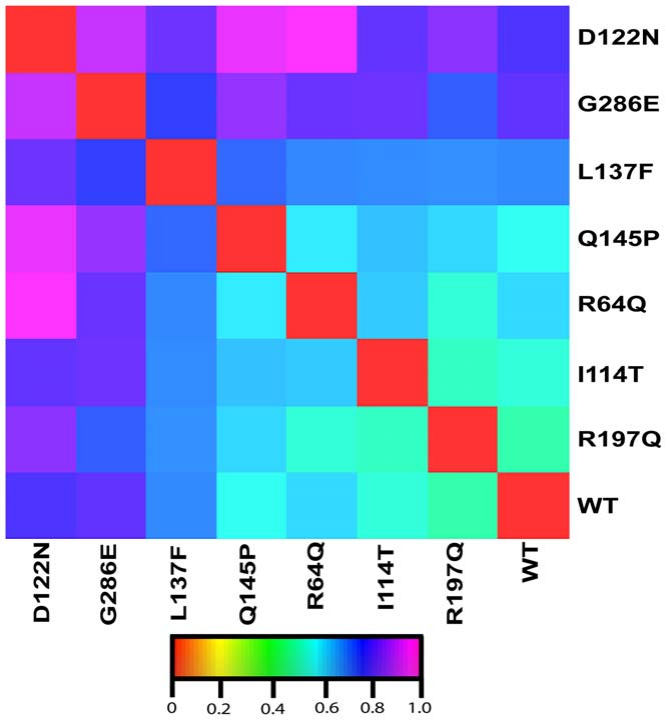
Protein interaction property similarity analysis (PIPSA) heat map of the WT and MT average structures. The electrostatic distance is calculated and represented by the Hodgkin similarity index [43]. The distances are color-coded from similar (red) to dissimilar (magenta). The last 10-ns averaged structures from one simulation of WT and MTs were used for the calculation. Similar behaviors were observed in all the simulations of WT and MTs.

The electrostatic surface potentials are important for protein structure and function. Mutations alter the protein surface electrostatics that lead to diverse effects on protein structure, including aggregation, folding and stability. Mutations in NAT2 often cause aggregation and/or reduced protein level because of degradation. To understand the effects of these mutations on protein surface charge property, we compared the electrostatic potential between the WT and MTs. The electrostatic potential of all MTs differed from that of the WT (Figure 7 and Figure S4). The difference in surface charge may result in aggregation and/or degradation on the MTs. Functional study of all MTs showed that reduced protein level may be caused by degradation. For R64Q, the change in surface charge property is the proposed mechanism for the aggregation [7], which may also apply to the other MTs.

### Probable ubiquitin substrate

Functional study of the MTs indicated that the protein level is reduced, probably by the proteasomal degradation pathway [46]. NAT2 contains 15 Lys residues, all of which are solvent exposed, and 9 are found in loops and flexible regions on the protein surface. In our simulations, 2 Lys residues, K13 and K18, located in the α1–α2 loop, and the other Lys residue, K184, located in the α7–β11 loop, showed increased solvent exposure (Table S1). From the differences in the WT and MT simulations, any one of these Lys(s) may serve as targets for the ubiquitin modification in the MT structure (Figure S5). However, further experiments such as mutational analysis or ubiquitination assays are needed to identify which Lys(s) is modified by ubiquitin.

### Conclusions

NAT2 polymorphism is one of most common in humans, and about half of all Caucasians possess the slow acetylator phenotype of polymorphisms [9], [47], [48], [49]. Because of the important role of NAT2 in the metabolism of arylamine, hydrazine drugs and many arylamine procarcinogens, understanding the structural basis of acetylation polymorphism is of interest. To analyze the effects of the SNPs on NAT2 structure and dynamics, we used multiple molecular dynamics simulations of WT and 7 MT structures, with 40-ns for each simulation. Although the polymorphisms are distributed in different regions, they have a cumulative effect on the catalytic triad, cofactor binding site, SBP, surface property and flexibility in different domains. The conformation of the catalytic triad residues, as well as the cofactor binding site and SBP, is altered severely in R64Q and D122N, which provides a possible structural basis for the reduced catalytic activity in these MTs. In comparison, conformation of the catalytic triad residues in the MTs L137F, Q145P and R197Q is slightly perturbed but in I114T and G286E is similar to that of the WT. For L137F, Q145P and R197Q, the cofactor binding site and SBP are severely distorted, which suggests a possible structural basis for the reduced catalytic activity, whereas I114T and G286E show a slightly distorted pocket, which suggests that these MTs can accommodate the substrate. Interestingly, the electrostatic potential of all MTs differs from that of the WT, which might affect their aggregation and/or degradation.

Although previous study described the structural features and functional effects of many NAT2 SNPs [10], the detailed analysis of structural and dynamics changes, such as changes in the catalytic triad, cofactor binding site and SBP, was not reported. Here, we give a detailed analysis of how a single amino acid substitution may cause major structural perturbations in functional sites situated away from the mutational site, which provides a better understanding of the role of NAT2 genetic polymorphism in bioactivation and detoxification of arylamine and hydrazine drugs and carcinogenesis. Further insight into the structure–function relationships of the SNPs of NAT2 may be revealed by investigation of other SNPs, such as R64W, E167K, K282T, and also C68Y and G51V, reported recently from a Brazilian population [50].

## Supporting Information

Figure S1 **Residues around the CoA and acetyl CoA.** (A) The hydrophobic residues around the CoA from the NAT2 crystal structure (PDB:2PFR). (B) The hydrophobic residues around the acetyl CoA from the modeled structure. This model was generated by the Oda *et al* [32] procedure. The hydrophobic residues are shown as green sticks and atoms of CoA and acetyl CoA are colored as C gray, N blue, O red, and H gray, P orange, S yellow.
(TIF)

Figure S2 **Overall Cα-RMSD of the wild-type (WT) and mutant (MT) structures with respect to the starting structures over 40-ns simulations.** Three independent simulations (black, red and green) of each protein were shown.
(TIF)

Figure S3 **Mutations in NAT2 did not affect the overall folding.** Snapshots from the last ns (40^th^) structures of three independent simulations (blue, pink and green) of each protein were superimposed.
(TIF)

Figure S4 **Electrostatic potential on the surface of WT and MTs.** Electronegative and electropositive charges are colored in red and blue, respectively.
(TIF)

Figure S5 **Potential sites of NAT2 ubiqutination.** NAT2 has 15 lysine residues distributed throughout the protein surface. In our molecular dynamics simulations, 3 lysine residues (K13, K18 and K184) located in unstructured regions of the protein become more solvent-exposed in all the MTs. Any one of these residues may be a substrate for ubiquitination. Cartoon diagram of R64Q from last ns (40^th^) structure is colored by domain (D1-wheat, D2-pink, ID-grey and D3-bluewhite). The 3 lysine residues are shown in stick representation.
(TIF)

Table S1 Solvent accessible surface area (SASA) of lysine residues calculated from the last 10-ns average structure of WT and MTs.
(DOC)
