# Supplementary figures and images for: Effects of Single Nucleotide Polymorphisms on Human N-Acetyltransferase 2 Structure and Dynamics by Molecular Dynamics Simulation
Source: PLoS One. 2011 Sep 29;6(9):e25801. doi: 10.1371/journal.pone.0025801 (PMC3183086; doi:10.1371/journal.pone.0025801)

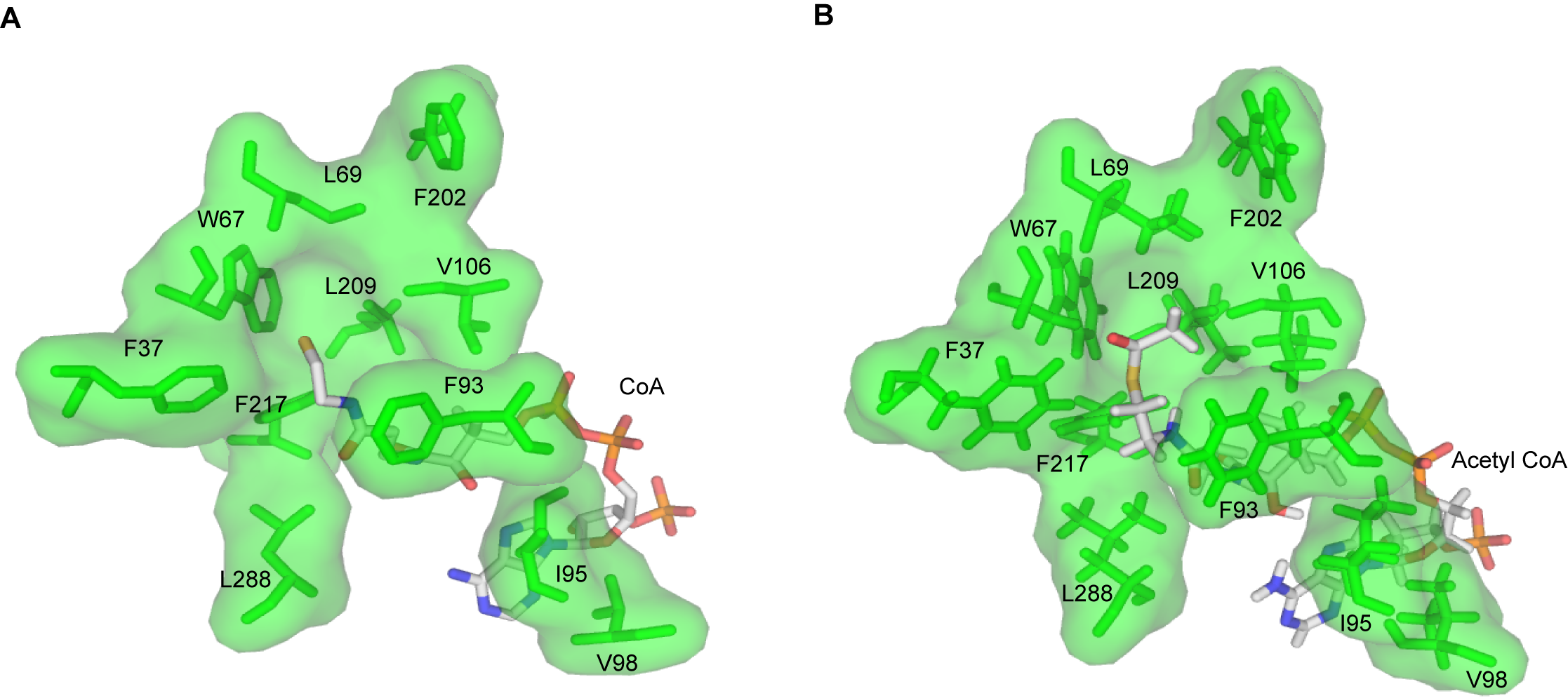

Supplement: Figure S1 — Residues around the CoA and acetyl CoA. (A) The hydrophobic residues around the CoA from the NAT2 crystal structure (PDB:2PFR). (B) The hydrophobic residues around the acetyl CoA from the modeled structure. This model was generated by the Oda et al [32] procedure. The hydrophobic residues are shown as green sticks and atoms of CoA and acetyl CoA are colored as C gray, N blue, O red, and H gray, P orange, S yellow. (TIF) [file pone.0025801.s001.tif]

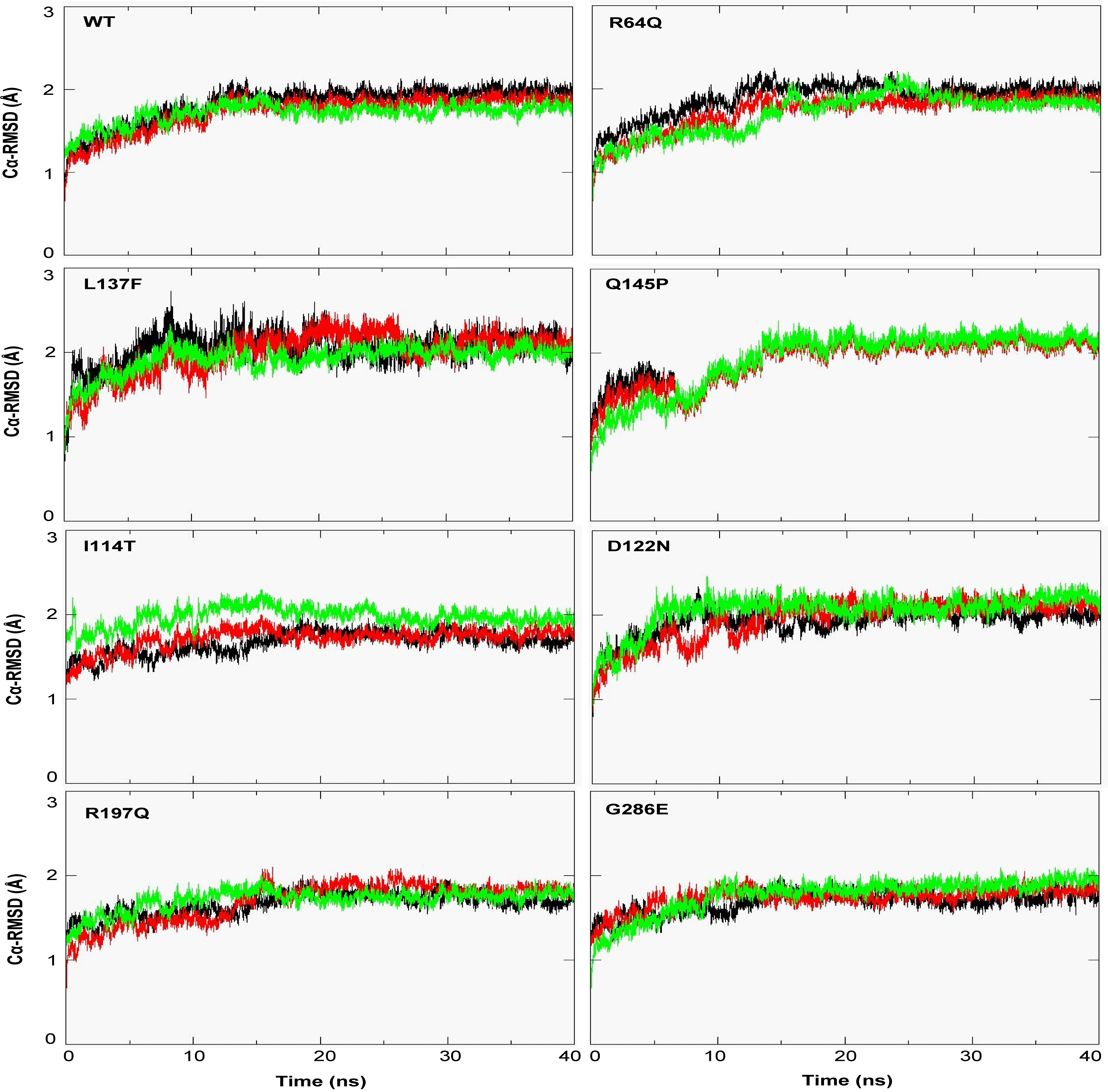

Supplement: Figure S2 — Overall Cα-RMSD of the wild-type (WT) and mutant (MT) structures with respect to the starting structures over 40-ns simulations. Three independent simulations (black, red and green) of each protein were shown. (TIF) [file pone.0025801.s002.tif]

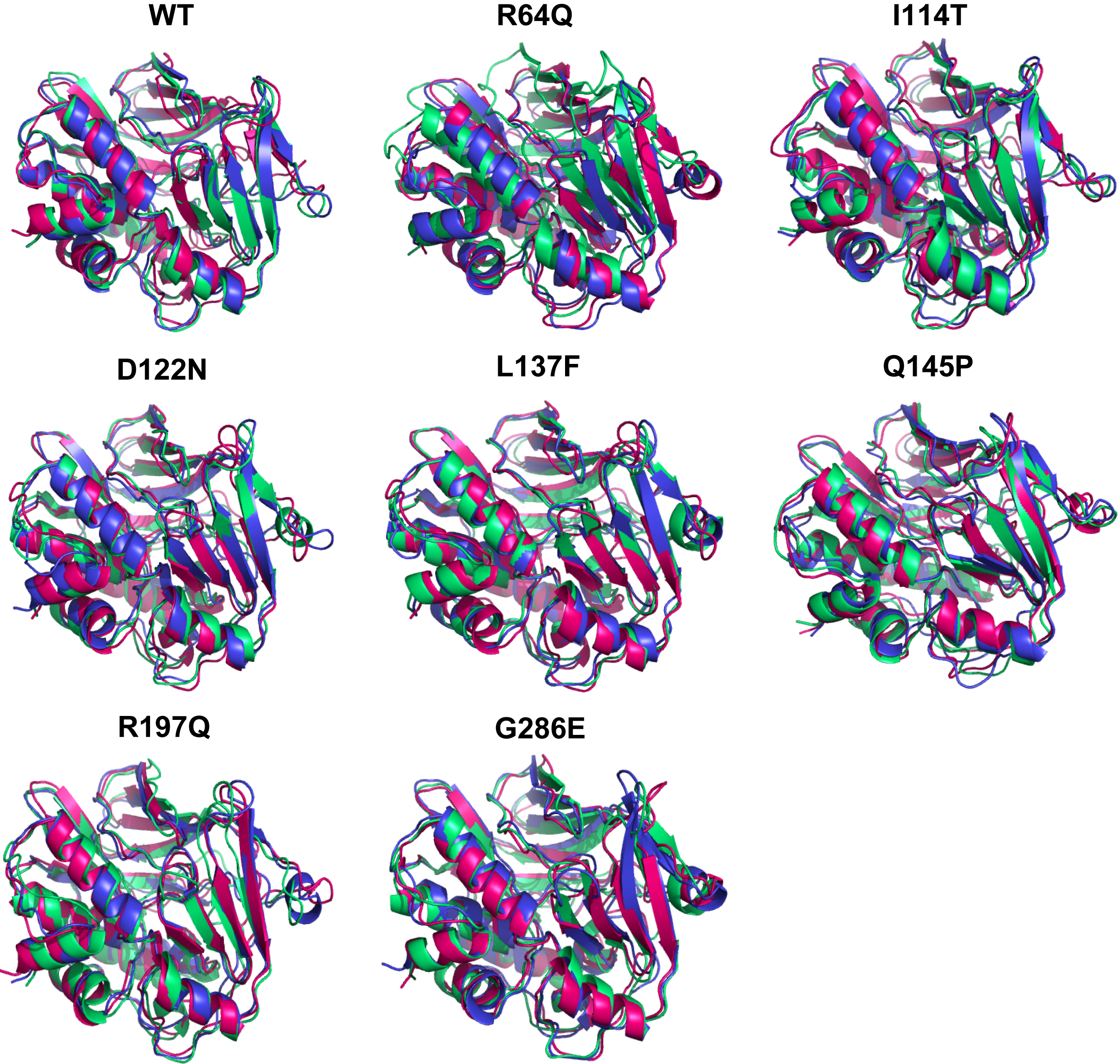

Supplement: Figure S3 — Mutations in NAT2 did not affect the overall folding. Snapshots from the last ns (40th) structures of three independent simulations (blue, pink and green) of each protein were superimposed. (TIF) [file pone.0025801.s003.tif]

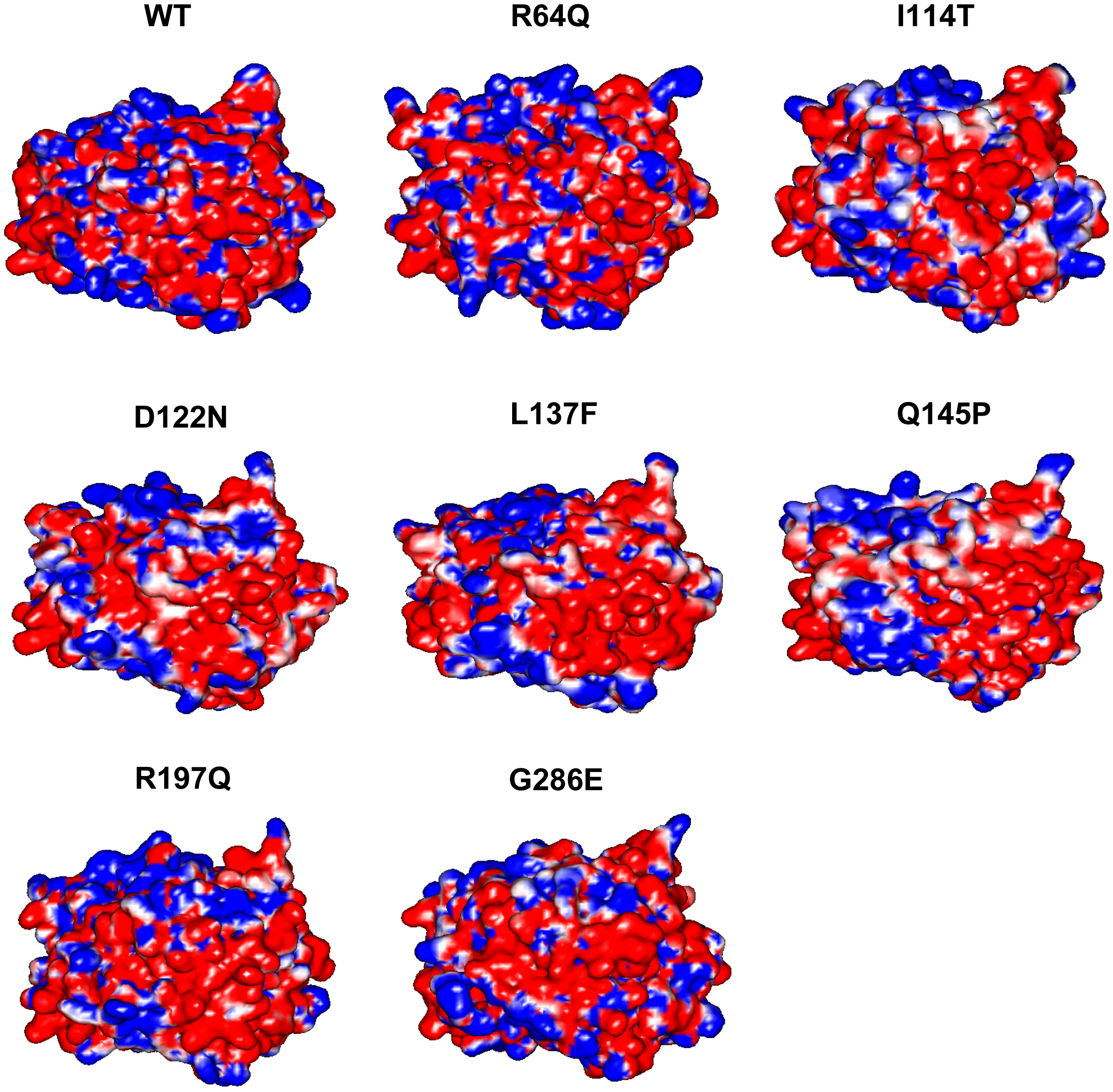

Supplement: Figure S4 — Electrostatic potential on the surface of WT and MTs. Electronegative and electropositive charges are colored in red and blue, respectively. (TIF) [file pone.0025801.s004.tif]

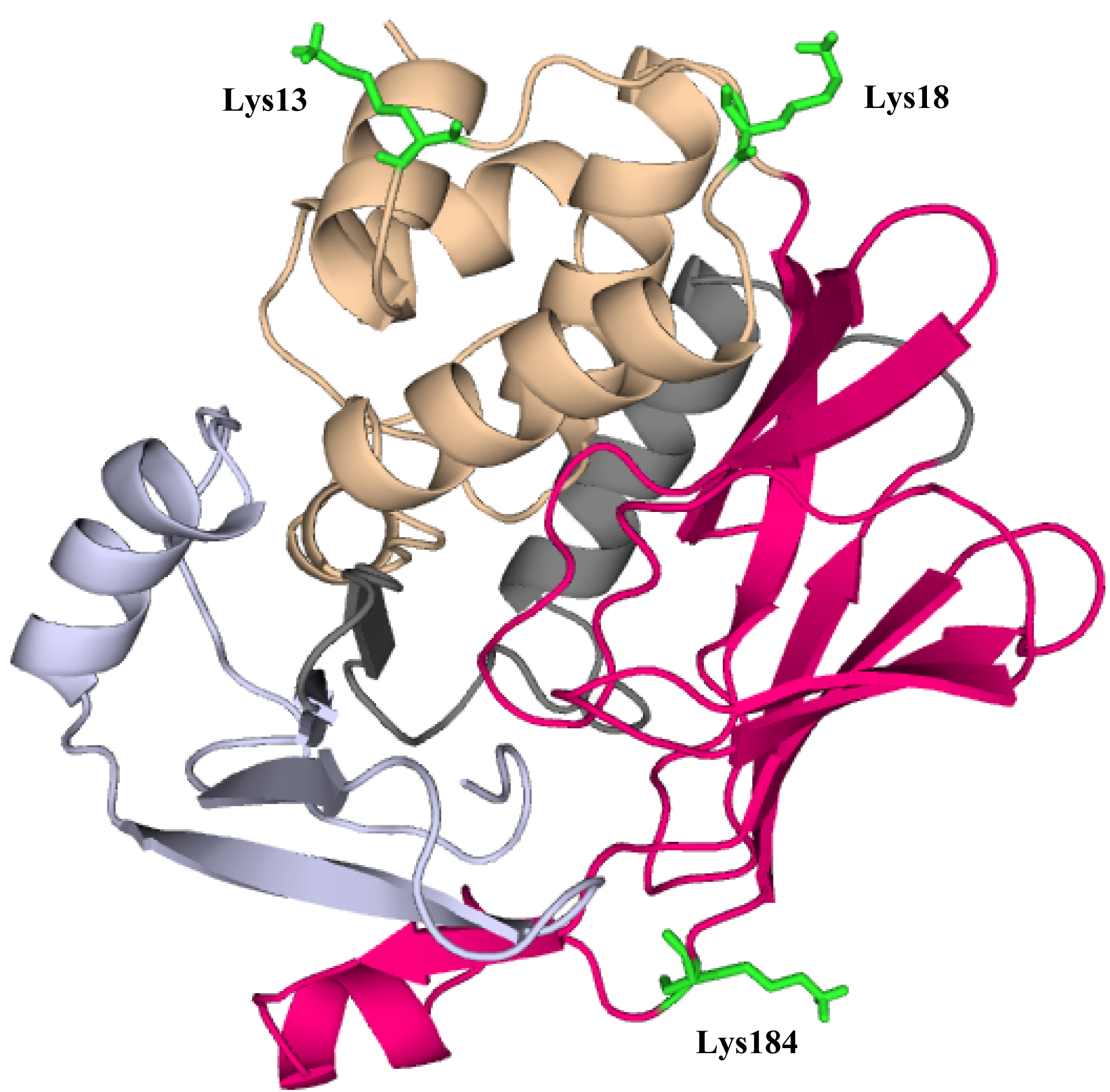

Supplement: Figure S5 — Potential sites of NAT2 ubiqutination. NAT2 has 15 lysine residues distributed throughout the protein surface. In our molecular dynamics simulations, 3 lysine residues (K13, K18 and K184) located in unstructured regions of the protein become more solvent-exposed in all the MTs. Any one of these residues may be a substrate for ubiquitination. Cartoon diagram of R64Q from last ns (40th) structure is colored by domain (D1-wheat, D2-pink, ID-grey and D3-bluewhite). The 3 lysine residues are shown in stick representation. (TIF) [file pone.0025801.s005.tif]
