# Supplementary material for: Effects of Single Nucleotide Polymorphisms on Human N-Acetyltransferase 2 Structure and Dynamics by Molecular Dynamics Simulation
Source: PLoS One. 2011 Sep 29;6(9):e25801. doi: 10.1371/journal.pone.0025801 (PMC3183086; doi:10.1371/journal.pone.0025801)
Supplement: Table S1 — Solvent accessible surface area (SASA) of lysine residues calculated from the last 10-ns average structure of WT and MTs. (DOC) [file pone.0025801.s006.doc]

**Table S1:** Solvent accessible surface area (SASA) of lysine residues calculated from the last 10 ns average structure of WT and MTs.

| LYS  Position | WT  SASA (Å^2^) | R64Q  SASA (Å^2^) | I114T  SASA (Å^2^) | D122N  SASA (Å^2^) | L137F  SASA (Å^2^) | Q145P  SASA (Å^2^) | R197Q  SASA (Å^2^) | G286E  SASA (Å^2^) |
| --- | --- | --- | --- | --- | --- | --- | --- | --- |
| **13** | **91.78** | **124.56** | **139.86** | **120.41** | **138.56** | **128.44** | **138.23** | **123.49** |
| **18** | **92.64** | **140.11** | **121.54** | **152.45** | **147.21** | **110.45** | **143.89** | **145.42** |
| 100 | 137.3 | 79.16 | 110.42 | 143.21 | 137.56 | 129.19 | 89.32 | 99.12 |
| *141* | *45.67* | *71.92* | *48.76* | *49.78* | *68.56* | *59.43* | *78.65* | *77.34* |
| *173* | *146.36* | *144.11* | *196.44* | *158.1* | *125.65* | *159.45* | *159.87* | *202.12* |
| 183 | 146.76 | 161.34 | 124.16 | 99.46 | 176.87 | 117.12 | 99.59 | 178.71 |
| **184** | **69.14** | **125.89** | **102.08** | **98.46** | **122.78** | **142.67** | **166.58** | **98.49** |
| 185 | 84.65 | 88.46 | 82.11 | 74.46 | 116.72 | 68.16 | 85.41 | 88.08 |
| *188* | 65.31 | 64.19 | 59.16 | 71.23 | 58.47 | 79.45 | 101.29 | 66.65 |
| *243* | *71.16* | *60.15* | *75.14* | *68.77* | *107.37* | *60.48* | *81.64* | *59.97* |
| 247 | 141.13 | 137.12 | 129.89 | 167.18 | 163.13 | 165.46 | 108.13 | 106.19 |
| *256* | *94.44* | *102.18* | *77.53* | *88.18* | *110.27* | *128.71* | *91.41* | *107.16* |
| *268* | *98.11* | *115.12* | *71.28* | *102.12* | *107.13* | *104.28* | *101.87* | *111.19* |
| 272 | 141.61 | 143.12 | 98.17 | 113.48 | 128.21 | 130.48 | 71.89 | 121.24 |
| 282 | 77.77 | 60.65 | 98.19 | 77.71 | 97.58 | 98.91 | 74.87 | 168.21 |

The lysine residues show higher solvent accessible surface area in all the MTs compared WT are shown in bold. The lysine residues which are located in the secondary structures are shown in italics.
